# Pleural puncture with thoracic epidural: A rare complication?

**DOI:** 10.4103/0019-5049.79898

**Published:** 2011

**Authors:** Rachna Wadhwa, Sandeep Sharma, Devadatta Poddar, Sujata Sharma

**Affiliations:** PGIMER and Associated Dr. R M L Hospital, New, Delhi, India

**Keywords:** Epidural catheter, post-operative pain, thoracotomy

## Abstract

Freedom from pain has almost developed to be a fundamental human right. Providing pain relief via epidural catheters in thoracic and upper abdominal surgeries is widely accepted. Pain relief through this technique not only provides continuous analgesia but also reduces post-operative pulmonary complications and also hastens recovery. But being a blind procedure it is accompanied by certain complications. Hypotension, dura puncture, high epidural, total spinal, epidural haematoma, spinal cord injury and infection are some of the documented side effects of epidural block. There are case reports eliciting neurological complications, catheter site infections, paresthesias, radicular symptoms and worsening of previous neurological conditions. Few technical problems related to breakage of epidural catheter are also mentioned in the literature. The patient had no sequelae on long term follow up even when a portion of catheter was retained. We present a case report where epidural catheter punctured pleura in a patient undergoing thoracotomy for carcinoma oesophagus.

## INTRODUCTION

Thoracic epidural catheters are placed to provide perioperative analgesia in a variety of thoracic and upper abdominal surgeries. Epidural catheters not only provide adequate analgesia but also reduce the incidence of post-operative respiratory complications[1] as well as hasten recovery in patients undergoing cardiac surgery.[2] Recent large prospective studies and meta analysis also concluded that although there is no significant reduction in mortality and overall major post-operative complications with epidural analgesia, they provide better control of pain postoperatively and significant reduction in respiratory complications.[3,4] Although various complications are associated with the procedure such as dura puncture, injury to spinal cord or nerves, formation of epidural haematomas and abscess, but its occurrence is rare. Pleural puncture is one of the technique-related complication, and a few such cases have been reported previously.[5–11]

## CASE REPORT

**Figure 1:**
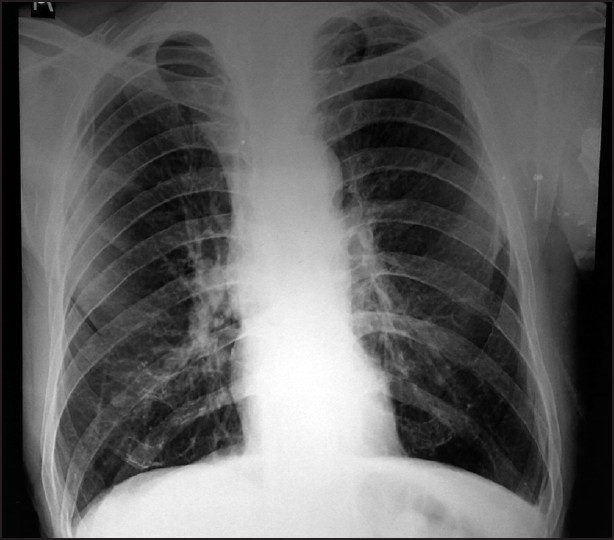
Pre op chest X-ray

**Figure 2:**
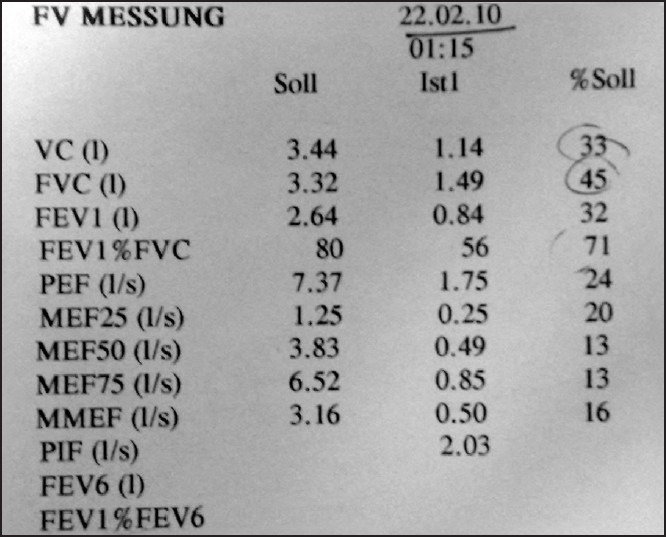
Numerical values of pulmonary function test

**Figure 3:**
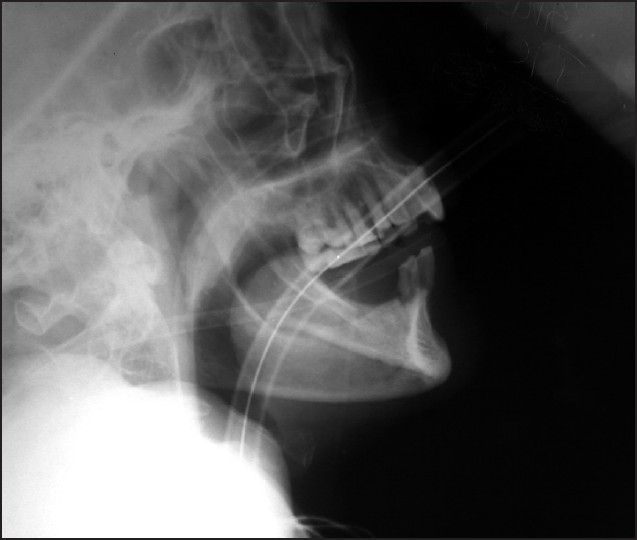
C-spine X-ray

A 62-year-old male (height 170cms, weight 45 kgs, Body Mass Index 15.5) was posted for elective oesophageal resection and reconstruction for carcinoma of the middle third of oesophagus. Patient was a chronic smoker and chronic alcoholic. He had a history of breathlessness at ordinary physical activity. Chest X-ray showed emphysematous lung fields [Figure 1]. In pulmonary function tests, there was severe obstructive airway disease pattern with observed FEV_1_ (Forced Expiratory Volume) being 30% only [Figure 2]. On examination neck flexion (10°) and extension (30°) were restricted [Figure 3]; mouth opening was three fingers with modified Mallampati Grade III. In the operating room, thoracic epidural via midline approach was planned before induction. Patient was placed in sitting position and under all aseptic precautions local anaesthesia (2 ml of 2% lignocaine) was infiltrated in thoracic T_6_-T_7_ intervertebral space. An 18-gauge epidural needle was inserted at an angulation of 40° with skin and epidural space was located in first attempt at 5 cm by loss of resistance to air technique. Epidural catheter was threaded very smoothly and then fixed at 9 cms at skin entry point. Epidural catheter was advanced 4 cms into the epidural space and was flushed with saline to check for its patency. Because of paucity of time both epidural test dose and analgesia was given after induction to this patient. There was no respiratory distress during and immediately after the procedure. Prior to induction, check laryngoscopy was done after nebulisation with lignocaine. It revealed Cormacke Lehane (IIA). While keeping the difficult airway cart ready, anaesthesia was induced with propofol 80 mg, fentanyl 50μg and vecuronium 5mg intravenously. After 3 min of intermittent positive pressure ventilation, intubation was done with 37 French left-sided double lumen tube. Anaesthesia was maintained with isoflurane 1 volume% in oxygen, vecuronium for muscle relaxation and fentanyl intravenously for analgesia. Epidural test dose of 3 ml of 1.5% lignocaine with 1:200,000 epinephrine was negative for intravascular or intrathecal injection.

**Figure 4:**
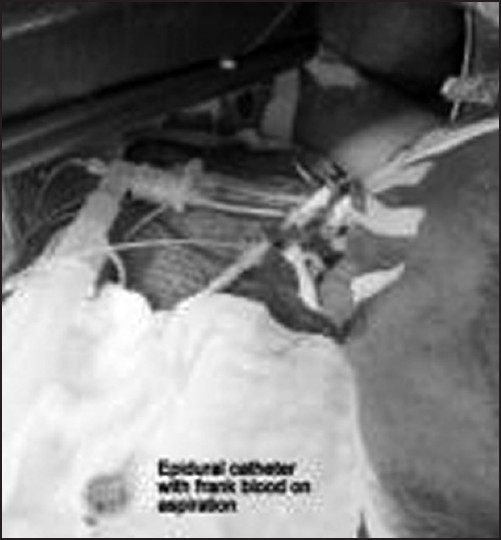
Blood in epidural catheter

**Figure 5:**
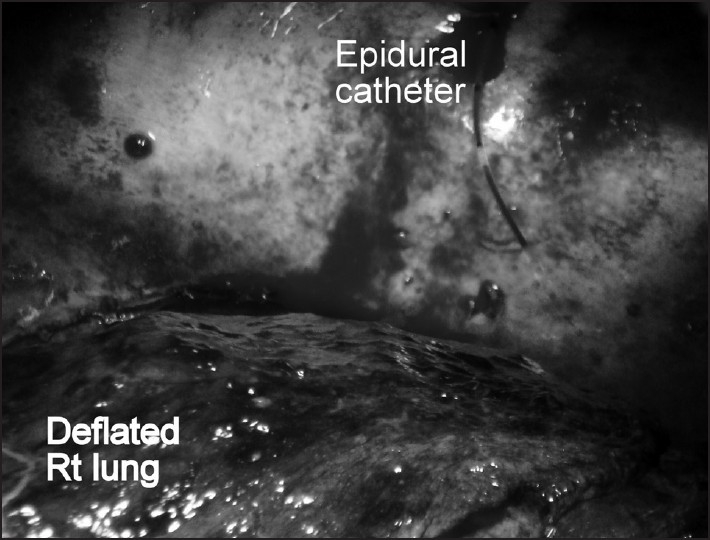
Catheter in thoracic cavity

Intraoperatively, epidural dose of bupivacaine 0.125% failed to cause decrease in heart rate and change in blood pressure. After 4 hours of laparotomy, thoracotomy was done on the right side. Repeated aspiration from epidural catheter during thoracotomy expressed frank blood, which continued even when catheter was withdrawn by 1 cm [Figure 4]. Epidural test dose was again repeated but it failed to show any changes in heart rate and electrocardiography. It was then suspected that epidural catheter might be misplaced and surgeons were asked to explore the epidural catheter, which was found lying in the right pleural cavity [Figure 5]. The epidural catheter was flushed with normal saline and removed very slowly. The tip of removed catheter was intact and the rest of the surgery was uneventful. Patient was transferred to intensive care unit for post-operative care and cardiopulmonary monitoring. He was discharged after 12 days from the Intensive Care Unit.

## DISCUSSION

Complications associated with epidural catheterisation are common. Even when performed with skilled hands, failure rate of thoracic epidural insertion is 8%.[6] Pleural puncture is a rare complication of thoracic epidural, which has been reported earlier. In most of the cases, complication occurred while introducing epidural catheter by the paramedian approach.[12] Midline approach, although difficult, is associated with less complication. Only two cases have been reported in which interpleural misplacement occurred while using midline approach.[6,9] In one of the cases it was difficult to locate the epidural space as the patient was obese (Body Mass Index- 36) and in the other even with no technical difficulties this complication was seen. These complications can be uneventful or can be life threatening in settings of haemothorax, tension pneumothorax. Our patient was at increased risk of this complication owing to thin built (Body Mass Index- 15.5), severe chronic obstructive pulmonary disease (emphysematous chest) and spine deformity. Thus, it is crucial to avoid and pay special attention to the possibility of misplacement during the performance of thoracic epidural catheterization. There were no respiratory symptoms after epidural catheter was placed in our patient probably because of small puncture by epidural needle, which might have got sealed by catheter itself. It is also advisable to put thoracic epidural catheter before induction to avoid risk of neurological damage unless patient is extremely nervous and uncooperative. Misplacement of epidural catheter may occur because of various reasons like spinal deformity, ligament calcification, poor positioning, landmarks etc.

Apart from the lean built our patient also had stiff neck and back with severely restricted movements, which explain the difficulty in feeling loss of resistance to air due to calcified ligament. It is also known that ligamentum flavum might fail to fuse in the midline at high thoracic level, and thus, it should not be relied on as a tactile landmark during thoracic epidural needle placement.[13,14]

Also, expression of frank blood after thoracotomy can be explained by collection of blood in para-vertebral spaces. This aspiration of blood with no effect to epidural test dose can be a sign for misplaced epidural catheter.

There is an instance by Amagasa *et al*. describing intercostal bleeding during thoracic epidural insertion in a patient undergoing VATS (video assisted thoracic surgery), but they experienced difficulty in placing it.[15]

To avoid such complications it can be concluded to refer to computer tomography scans or ultrasonographic-derived measurement of depth from skin to epidural space, assessment of preoperative sensory level of anaesthesia for confirmation of correct placement of epidural catheter especially in patients with spine deformity. It is also advisable to give epidural block and check for the level of block prior to induction in order to confirm the correct placement. Other important recommendations are as follows: (1) Do not thrust catheter forcefully, (2) Avoid repeated punctures with tuohy needle in one space, (3) Try to achieve optimal position of patient before attempting regional anaesthesia, and (4) Plan median or paramedian approach according to the patient.[16]

In contrast to our immediate removal of the misplaced epidural catheter there are studies in which this intrapleural catheter was used to provide peri-operative analgesia with good results.[17]

This case report highlights the facts pertaining to problems of thoracic epidural and different ways to manage them for the benefit of the patient.
